# Mosquitoes in small urban spaces: identification of blood meals and flight distances of engorged females in the southern Great Plains of the United States

**DOI:** 10.1093/jme/tjaf105

**Published:** 2025-08-23

**Authors:** Brandon E Henriquez, Scott R Loss, Bruce H Noden

**Affiliations:** Department of Entomology and Plant Pathology, Oklahoma State University, Stillwater, OK, USA; Department of Natural Resource Ecology and Management, Oklahoma State University, Stillwater, OK, USA; Department of Entomology and Plant Pathology, Oklahoma State University, Stillwater, OK, USA

**Keywords:** small urban areas, wildland–urban interface, blood meal, *Aedes albopictus*, *Anopheles quadrimaculatus*

## Abstract

Vector-borne disease transmission can only occur when host(s), vector(s), and pathogen(s) interact in a given environment. While many studies have focused on these interactions in large urban areas, there is a need for habitat-focused studies in small urban areas where human populations are often close to wildlife and livestock. The aim of the current study was to identify the bloodmeal sources of mosquitoes in a small urban area in the southern Great Plains of the United States. Using 2 trap types, bloodmeals from 12 different hosts were detected, and the most frequently detected bloodmeal hosts were white-tailed deer, cow, and horse. The known locations of livestock at each site made it possible to identify the nearest location where mosquitoes could have fed on cows, horses, and alpacas, and we demonstrated that mosquitoes could fly distances between 200 m and 1.2 km from the bloodmeal host to the resting trap location within 30 h after taking blood. This study highlights the opportunities that are available within small urban areas to discover important host–vector relationships.

## Introduction

Vector-borne disease transmission can only occur when host(s), vector(s), and pathogen(s) interact with specific biotic and abiotic factors in a given environment (ie the “nidus of infection” Reisen 2010). In any vector-borne disease system, the variability of host and vector abundance and diversity influences vector–host interactions, and therefore, the potential for pathogen transmission to occur across a landscape (Kilpatrick et al. 2006, Gottdenker et al. 2014, Hassell et al. 2017, Guo et al. 2019). Because of this, it is imperative to identify potential hosts, the diversity of vectors, and critical characteristics of the landscape, to predict where disease transmission may occur. Habitat-based approaches are often used to provide critical understanding of how the components of the nidus connect (Reisen 2010). Fine-scale studies measuring how individual mosquito species utilize their habitat have been carried out in different regions of the United States (Bidlingmayer and Hem 1981, Lothrop and Reisen 2001, Thiemann et al. 2011, Reiskind et al. 2017). These studies not only provide unique insights into mosquito behavior but they also provide a new impetus for addressing vector-borne disease transmission.

An important component of the “nidus” of vector-borne disease is identifying relationships between mosquito vectors and particular hosts within specific land cover types. The majority of US-based studies involving the identification of mosquito blood-meal hosts are specific to one particular disease system (WNV, EEE) (Molaei et al. 2007, 2009, Thiemann et al. 2011), a specific mosquito genus (Mackay et al. 2010, Kahl et al. 2021) or species (Kent et al. 2009, Reisen et al. 2013, Little et al. 2021), or a particular land cover type, particularly large urban or rural areas (Thiemann et al. 2017, Zettle et al. 2022, Yitbarek et al. 2023). This focus on specific pathogens, hosts, mosquito species, or land cover type may address certain research questions, but our general knowledge of host interactions for most mosquito species across much of the United States remains understudied (Santiago-Alarcon 2022). Of host blood-meal studies published in the United States since molecular methods (PCR) have been used to identify hosts, less than one-third have published the host bloodmeals from most of the blood fed mosquitoes collected (Santiago-Alarcon 2022). These “all host—all mosquito” blood-meal studies have involved large urban areas (Goodman et al. 2018, Mann et al. 2020), rural forests or state land (Burkett-Cadena et al. 2011, Thiemann et al. 2017), zoos (Tuten et al. 2012, Briggs et al. 2023), and statewide surveillance (Apperson et al. 2004, Molaei et al. 2008).

One under-represented land cover type is small urban areas in the United States. Small urban areas, which are defined as cities containing greater than 5,000 persons and less than 50,000 persons (Federal Registrar 2022). Small urban areas provide unique environments in which to study host–vector interactions. Often surrounded by farms with livestock (McCleery 2010), a high percentage of the inhabitants in small urban areas live at the interface where undeveloped wildland meets housing developments [ie the wildland–urban interface (WUI) (Radeloff et al. 2005)]. WUI-based studies have mainly explored fire risk (Radeloff et al. 2018), exposure to vector-borne disease in large urban areas (Bisanzio et al. 2020, Kache et al. 2022) and wildlife interactions (Bradley and Altizer 2007, Niesner et al. 2021). The presence of people and livestock in the WUI of small urban areas provides a unique context to explore host–vector relationships within the context of nidality. The close proximity of housing developments and schools to pastures containing cows and horses could provide a better understanding of host–vector interactions. These spatial relationships between animals and people provide an environment also to potentially estimate mosquito post-bloodmeal flight distances (post-prandial movement) as it applies to risk of exposure and potential for pathogen dissemination. This information, then, could be used to clarify the risk of exposure of people living in these small urban areas to vector-borne pathogens (eg WNV).

Taking advantage of the WUI within a small urban area in the southern Great Plains, the aim of the current study was to identify the bloodmeal sources of mosquitoes. In the process of analyzing the data, we also identified the locations of specific animals that likely had been fed upon and calculated a minimum estimate of the post-prandial movement for various mosquito species. The outcomes of this study will provide valuable information for public health and land management programs throughout the Great Plains.

## Methods and Materials

### Study Locations

This study was conducted in a small urban area with low and moderate levels of development and population density in central Oklahoma (Federal Registrar 2022). In Oklahoma, 88% (66/75) of the “urban areas” are considered “small urban areas” with populations below 50,000 (Oklahoma Demographics 2025). Within most small urban areas in central Oklahoma, an invasive native tree, *Juniperus virginiana* L. (eastern redcedar [ERC]) has encroached into backyards and parks (Engle et al. 2008), providing a natural habitat for trapping mosquitoes in urban areas throughout Oklahoma (Noden et al. 2021). For this study, we chose collection locations in city parks and private land that were easily accessible with <1 ha thickets of concentrated trees, most consisting of ERC and averaging greater than 3 m in height. Once chosen, permissions were sought after which we visited each site to establish locations within the ERC trees where traps could be placed. Some sites were forested so we were able to accommodate more traps while others were lines of trees sheltering grassland. During the sampling season, 15 resting traps were set up at 4 established trapping locations: Highland Park (6 traps), Sangre Ridge (2 traps), Briarcreek (3 traps), and Whittenberg Park (4 traps). To augment mosquito collections and mosquito diversity, especially blood-fed mosquitoes, we set out 2 CDC light traps (Bioquip, Rancho Dominguez, CA, USA) for one-night collections on 2 occasions (8 and 23 July 2021) at each trapping location, resulting in 16 additional trapping nights.

### Trapping Protocol

Before collections began, resting (bucket) traps (Burkett-­Cadena et al. 2011) were placed at the 4 trapping locations and remained in place for the entire sampling season. Because our objective was to collect as many blood-fed mosquitoes as possible, we placed the resting traps in each trapping location within 10 to 20 m of each other. Collections began on 23 June 2021 through 30 July 2021 with resting traps visited 2 to 3 times per week before 10:00 AM depending on the weather conditions. Resting traps were placed inside the lower branches of ERC trees with the openings flush with ERC tree foliage. At each visit, all resting traps and a 1 m area of the surrounding foliage were aspirated with InsectZooka Field Aspirators (Bioquip, Rancho Dominguez, CA, USA). To attract host-seeking mosquitoes for 2 additional nights, we hung CDC light traps (Bioquip, Rancho Dominguez, CA, USA) with containers containing dry ice from ERC branches, 1.5 m from the ground. Lights were removed to reduce nontarget insect collection. The CDC trap locations were set up prior to 4:00 PM and collected the following morning by 10:00 AM.

### Mosquito Identification

Mosquitoes collected from resting traps in Insectzooka collection cups (Bioquip, Rancho Dominguez, CA, USA) as well as the CDC light traps collection cups were labelled by date, trapping location, trap type, and trap number. At the lab, mosquitoes were immediately sorted by feeding status to prevent degradation of host DNA in the bloodmeal (Coulson et al. 1990). Mosquitoes containing bloodmeals were individually stored in 1.5 ml clear microtubes and stored in a −80 °C deep freezer until bloodmeals could be extracted (Molaei et al. 2008). All other mosquitoes were stored at −20 °C until they could be identified using established keys (Darsie and Ward 2005).

### Bloodmeal Identification

The process of blood-meal identification began by removing the blood-engorged abdomen from the rest of the mosquito body in a drop of PBS buffer on a glass microscope slide (Mann et al. 2020). If the abdomen burst, the blood and PBS was pipetted into tubes pre-filled with 200 µl of PBS. Bloodmeal DNA was extracted following the manufacturer’s protocols (DNeasy 96 Blood and Tissue Kit, Qiagen, Valencia, CA, USA) (Thiemann et al. 2012). Extracted DNA was stored at −20 °C until PCR amplification. Blood meal hosts were identified using PCR amplification and DNA sequencing using a fragment of either the vertebrate mitochondrial genes cytochrome oxidase 1 (*COI*) or cytochrome b (*CytoB*). All samples were initially screened with a *COI* primer cocktail consisting of forward primers (*VF1 t1*, *VF1d t1*, and *VF1i t1*) and reverse primers (*VR1d t1*, *VR1 t1*, and *VR1i t1*) each mixed at a ratio of 1:1:2 (Ivanova et al. 2007). If the initial screening produced no useable sample, samples were rerun with *CytoB* primers focused on specific avian (Molaei et al. 2006), mammalian (Molaei et al. 2006), and reptilian/amphibian (Cupp et al. 2004) sequences. As occasionally happens with blood fed mosquitoes in Oklahoma (Henriquez et al. 2025), if the *COI* primer cocktail produced a sequence that matched for *Anaplasma* spp., mammalian specific *CytoB* primers (Molaei et al. 2006) were used to determine blood meal host while the *16S* rRNA primers (*PER1/PER2*) (Munderloh et al. 1996) were used to determine the *Anaplasma* species. PCR samples were then run in a 2% agarose gel containing ethidium bromide in a 1x TBE buffer and visualized under ultraviolet light. All amplicon-positive samples were extracted from the gels using the QIAquick Gel Extraction Kit (Qiagen) and bidirectionally sequenced at the Oklahoma State University Core Facility to confirm positivity and identify animal or bacterial species. We compared resulting consensus sequences with GenBank submissions using default conditions on NCBI BLAST (highly similar sequences [megablast]) where the highest % sequence identity was used to determine species identity.

### Post-Feeding Flight Distances

As we determined the host identities for each blood-fed mosquito, we noted site-specific patterns that made it possible to identify the minimum distance to the closest pasture or pen where the mosquitoes could have fed on a particular host species. The uniqueness of working in a small urban community made it possible to identify where specific animals were pastured within the community by walking/driving the area or by talking with homeowners nearby. Post-prandial flight studies have identified that mosquitoes fly, on average, considerably less than 500 m after taking a blood meal (Greenberg et al. 2012, Heym et al. 2019, Martínez-de la Puente et al. 2020, Hernandez-Colina et al. 2021). Using this conservative measurement, we decided that if there was more than one of the same animal species in different pastures within a 1 km perimeter around the collection site, we assumed the mosquito could have come from any of the pastures, and thus, we did not generate distance measurements. We made sure that only mosquitoes for which each bloodmeal could be linked to unique animals in only one possible pasture/pen near the collection area were included in the post-flight study group. We used Google Earth to estimate the minimal distance flown by recently blood fed female mosquitoes. Because the resting traps were spread within 10 to 20 m in each collection area, we did not attempt to link the animal site with specific resting traps, choosing instead we recorded location coordinates from the middle of where resting traps were located. We took the minimal distance based on the edge of the pasture where the unique animal was located and, using our local knowledge, estimated the minimal straight-line distance that mosquitoes would navigate in a given location. We recognize that post-fed mosquitoes may have utilized other flight patterns to return to the collection location in a given area, but our methodology was to take the most conservative approach to capture the straight-line distance of the most obvious route.

## Results

### Mosquito Collections

A total of 925 mosquitoes consisting of 23 species were collected at 4 suburban trapping locations between 23 June and 30 July 2021 (Supplementary Table S1). The majority (591, 63.9%) of mosquitoes were collected on 2 nights using the CO_2_-baited CDC light traps. Of the species collected, most *Aedes trivittatus* (Coquillett, 1902) (83%), *Psorophora cyanescens* (Coquillett) (77%), *Ps. ferox* (von Humboldt) (83%), and *Ps. longipalpus* Randolph & O'neill (81%) were collected at Highland Park while most anophelines (*Anopheles quadrimaculatus* Say [92.6%], *An. punctipennis* [Say] [74%]) and *Culex erraticus* (Dyar & Knab) (60%) were collected in Briarcreek. Notably, an *An. perplexens* Ludlow was collected at Whittenberg. Of the medically important mosquitoes, the most common species collected was *An. quadrimaculatus* (190) followed by *Cx. erraticus* (183), *Ae. albopictus* (Skuse) (140), *Ps. columbiae* (Dyar & Knab) (36), *Cx. pipiens* L. (23), and *Cx. tarsalis* (7).

### Bloodmeal Identification

A total of 95 blood-fed mosquitoes from 8 species were collected during the study period (Table 1). The majority of blood-fed mosquitoes (83, 87.4%) were collected using resting traps with the majority (69, 72.6%) consisting of *An. quadrimaculatus*. We confirmed the host specificity for 72 (75.8%) of the blood-fed mosquitoes collected (Table 2). Of 12 different hosts detected, the most frequently detected blood meals were white-tailed deer (*Odocoileus virginianus* [Zimmermann] [WTD]) (23, 31.9%), by cow (*Bos taurus* L.) (19, 26.4%) and horse (*Equus ferus caballus* L.) (13, 18.0%). Only one avian bloodmeal, a mourning dove (*Zenaida macroura* L.), was detected in a *Ps. columbiae*. We also identified unique hosts such as an armadillo (*Dasypus novemcinctus* L.) (1), a cat (*Felis catus* L.) (1), eastern Cottontail rabbit (*Sylvilagus floridanus* [J.A. Allen]) (3), opossum (*Didelphis virginiana* [Kerr]) (1) and an alpaca (*Lama pacos* L.) (1) in addition to 2 bloodmeals (*An. quadrimaculatus* [1] and *Ae. albopictus* [1]) from humans (*Homo sapiens* L.). We identified 4 (5.6%) mosquitoes (*An. quadrimaculatus* [3]) and *Ps. columbiae* [1]) at different sites that had fed on WTD infected with *Anaplasma platys* Dumler (Genbank Accession no. PQ892083 [100% identity]).

**Table 1.** Total number of blood-fed mosquitoes collected between June and July 2021 at 7 trapping locations in central and western Oklahoma

	Location					Trap type		
Species	Briarcreek	HighlandP	SangreR	Whittenberg	Total	CDC	Bucket	Total
*Ae. albopictus*	1	0	0	0	**1**	1	0	**1**
*An. quadrimaculatus*	61	2	6	0	**69**	0	69	**69**
*An. punctipennis*	3	0	0	0	**3**	0	3	**3**
*Cx. erraticus*	8	2	0	0	**10**	2	8	**10**
*Cx. nigripalpus*	2	0	0	0	**2**	0	2	**2**
*Cx. pipiens*	1	0	0	1	**2**	1	1	**2**
*Ps. columbiae*	0	0	0	5	**5**	5	0	**5**
*Ps. ferox*	0	1	0	0	**1**	1	0	**1**
Unknown	0	2	0	0	**2**	2	0	**2**
Total	76	7	6	6	**95**	12	83	**95**

**Table 2.** Total number of blood-fed mosquitoes that fed on specific hosts between June and July 2021 in trapping locations in central and western Oklahoma

Mosquito species	Total no.	Cow	Horse	Alpaca	WTD	Human	Sheep	Raccoon	Opossum	Armadillo	Cat	Rabbit	Mourning dove
*Ae. albopictus*	**1**	–	–	–	–	1	–	–	–	–	–	–	–
*An. punctipennis*	**3**	–	–	–	2	–	1	–	–	–	–	–	–
*An. quadrimaculatus*	**47**	15	11	1	13	1	5	–	–	–	–	1	–
*Cx. erraticus*	**9**	1	1	–	4	–	–	–	1	–	1	1	–
*Cx. nigripalpus*	**2**	–	–	–	2	–	–	–	–	–	–	–	–
*Cx. pipiens*	**2**	–	–	–	1	–	–	–	–	–	–	–	1
*Ps. columbiae*	**5**	3	1	–	1	–	–	–	–	–	–	–	–
*Ps. ferox*	**1**	–	–	–	–	–	–	–	–	–	–	1	–
Unknown	**2**	–	–	–	–	–	–	1	–	1	–	–	–
Total	**72**	19	13	1	23	2	6	1	1	1	1	3	1

### Flight Distance

At the Briarcreek site, we identified 19 *An. quadrimaculatus* that had flown varying minimum distances from their presumed feeding site on sheep (357 m), cows (357 m), and alpaca (1,265 m) to the trapping location (Table 3; Supplementary Fig. S1A and B). At Whittenberg Park, we identified 2 *Ps. columbiae* that had flown a minimum of 593 m from their presumed feeding site on cows into the ERC encroached area where the resting trap was located (Supplementary Fig. S2). At the Sangre Ridge site, we identified an *An. quadrimaculatus* that flew a minimum of 502 m, after having fed on a horse (Supplementary Fig. S3). At the Highland Park site, a *Cx. erraticus* and an *An. quadrimaculatus* flew at least 194 m after feeding on a horse which was housed next to an industrial business complex (Supplementary Fig. S4).

**Table 3.** Minimum post-prandial flight distances from closest known mammalian hosts by mosquitoes collected in eastern redcedar forests in Stillwater, OK

Site	No. collected	Mosquito	Host	Distance, m	Trap
Briarcreek	5	*An. quadrimaculatus*	Sheep	357	Bucket
	13	*An. quadrimaculatus*	Cattle	357	Bucket
	1	*An. quadrimaculatus*	Alpaca	1,265	Bucket
	1	*Cx. erraticus*	Cow	357	Bucket
Whittenberg Park	2	*Ps. columbiae*	Cattle	593	Light
Sangre Ridge	1	*An. quadrimaculatus*	Horse	502	Bucket
Highland Park	1	*Cx. erraticus*	Horse	194	Light
	1	*An. quadrimaculatus*	Horse	194	Bucket

## Discussion

Results from this study demonstrate that mosquito species of medical importance in small urban areas in the southern Great Plains of the United States are actively feeding on common livestock species and flying long distances to rest in small, forested areas soon after taking blood-meals. Eight species of mosquitoes, trapped at 4 collection locations, contained bloodmeals from 12 different hosts, most of which were white-tailed deer, cows, and horse. By linking pastures containing unique animals with collection locations, we calculated minimum estimates of post-feeding flight for a sub-set of mosquitoes, and demonstrated that mosquitoes could fly distances of between 200 m and 1.2 km from the bloodmeal host to the resting trap location within 30 h after taking blood.

The unique setting of the study provided an opportunity to learn more about the hosts that mosquitoes feed on in small urban areas in the southern Great Plains. Although 12 different hosts were detected among the 8 species of blood-fed mosquitoes collected, most of the detected bloodmeals (76%) were from white-tailed deer, cattle, or horses. These mammals are readily available in pastures and in a variety of woody cover types including areas experiencing ERC encroachment throughout urban areas in the southern Great Plains (McAninch 1995, Bidwell et al. 2017, Noden and Dubie 2017, Hiney 2018). While some mosquitoes are host specific, most are catholic feeders, broadly mammalophilic or generalists (Washino and Tempelis 1983, Chaves et al. 2010), and feed on whatever is available. In this study, mosquitoes fed almost exclusively on mammals with only one avian bloodmeal detected (a mourning dove). This is interesting as several studies report that up to 75% of the hosts fed on by *Culex* species during June–August are avian (Kent et al. 2009, Burkett-Cadena et al. 2011, Reisen et al. 2013). Yet, in the same time period for the current study, 92% of the blood-fed *Culex* fed on mammals in this urban area. While only one mosquito had fed on an avian host, the detection of WNV-infected mosquitoes in these same communities by other studies indicate that they are feeding on birds as well (Noden et al. 2021, Maichak et al. 2022, Henriquez et al. 2025).

The high number of bloodmeals identified from WTD in mosquitoes collected in ERC encroached areas provided the detection of a unique tick-borne pathogen. In the process of screening, we identified 4 mosquitoes from 2 different species, *An. quadrimaculatus* (3) and *Ps. columbiae* (1) from 2 locations that had fed on WTD and also were positive for *Anaplasma platys*. The detection of tick-borne bacterial species in mosquito bloodmeals was not surprising and is commonly used in xenosurveillance programs (Fernández de Marco et al. 2016, Fauver et al. 2017). In our study, the *Anaplasma platys* detected is transmitted by *Rhipicephalus sanguineus* (Latreille) ticks to WTD and dogs and is a significant canine pathogen in this region (Little et al. 2021). It was quite unexpected, however, that our initial screening of these mosquito blood meals amplified bacterial DNA instead of DNA from the mammalian host on which the mosquito had fed. However, universal COI primers occasionally amplify bacterial DNA (Hintikka et al. 2022). This likely demonstrated that the deer hosts had active infections with this bacterial species at the time they were fed upon by the mosquito. While this is the first report of this pathogen in WTD in Oklahoma, *Anaplasma platys* has been reported from WTD in Alabama and Texas (Rankins et al. 2017, Yu et al. 2020). This finding becomes more important as more studies report the possible zoonotic risk of *A. platys* in humans (Maggi et al. 2013, Arraga-Alvarado et al. 2014, Breitschwerdt et al. 2014).

While many studies have evaluated flight distances for unfed mosquitoes (Eyles et al. 1945, Ciota et al. 2012, Hamer et al. 2014, Medeiros et al. 2017), ERC-encroached sites in small urban areas provided a unique setting in which to study post-prandial movement of mosquitoes. Many of these urban areas in the Southern Great Plains are surrounded by grassland containing cows and horses as well as alpacas, regardless of the size of the town (Guiling et al. 2017). These pastures are usually small hobby farms owned and maintained by people who work in the nearby urban area and are stocked with livestock species like cattle, sheep, and horses. Due to this proximity to known locations of livestock, it was possible to estimate the minimum distance flown by recently blood fed female mosquitoes. These estimates could only be made for livestock as it was not possible to accurately identify the precise locations of the deer that were fed on by mosquitoes because deer are free-ranging and not confined to specific areas. (Gee et al. 2011) Post-prandial movement studies have utilized marking [eosin (Le Prince and Griffitts 1917), fluorescent dyes (Russell et al. 2005) or radioactive isotopes (Zhang et al. 2014)] to track mosquitoes after feeding and others have used the unique settings of zoos to estimate distances to the locations of the unique animals detected in mosquito blood meals (Greenberg et al. 2012, Martínez-de la Puente et al. 2020, Hernandez-Colina et al. 2021). Despite not involving specific marking techniques, our results were similar to those reported by others from different countries. Studies from zoos have reported minimum post-prandial distances in a variety of species up to 100 to 770 m (Greenberg et al. 2012 [NM, USA], Heym et al. 2019 [Germany], Martínez-de la Puente et al. 2020 [Spain], Hernandez-Colina et al. 2021 [UK]) while marking studies have reported distances up to 240 m (Russell et al. 2005 [Australia], Zhang et al. 2014 [China]). Conversely, experiments on blood-fed *Culex pipiens* using a “flight mill” demonstrated that mosquitoes are able to fly at least 1,500 m within 1 d after taking a blood meal (Clements 1955), while blood-fed *Ae. sollicitans* (Walker) have been recorded over 3,000 m from their bovine host (Schaefer and Steelman 1969 [LA, USA]).

While there is no way to know the exact flight route used by each mosquito between the point of blood-meal and resting traps, the possible flight pattern used after taking the blood-meal is important to consider. In addition to long post-prandial movement distances, the flights had to occur across diverse terrain within the first 30 h after taking the blood meal or the DNA would have been too degraded to be useful for end-point PCR (Oshaghi et al. 2006). Post-prandial studies in Ghana demonstrated that, after feeding, *Anopheles coluzzi* Coetzee & Wilkerson remained within 50 m of the host for the first 7 h, after which they dispersed up to 250 m in 60 h (Orsborne et al. 2019). Although further research would be needed to confirm the possibility, this observation suggests that females in the current study could have rested for a few hours near the feeding site after they imbibed almost 3 times their body weight (Clements 1992) before initiating long flights to the resting traps. For the longest flight corridor involving the alpaca blood-meal, the post-prandial movement of the *A. quadrimaculatus* female to the trapping location could have traversed over 1 km of blacktopped road lined with trees, streams, and diverse vegetation, including a separate line of dense ERC trees. This would not be unusual for *An. quadrimaculatus*, which has been recorded as flying 940 m after feeding, 240 m of which was across a river (Le Prince and Griffitts 1917). Other corridors between feeding and trapping locations in the current study possibly included 500 m of open suburban blacktop lined with 23 residential yards, as well as navigation of nearly 600 m involving the crossing of 2 to 3 residential yards and a 200 m stretch of open grassland. Given the potential predation risk by birds or insects during daylight hours, it is highly likely that the blood-fed mosquitoes travelled at night (Bidlingmayer 1971) but the risk of a heavily blood-fed mosquito being detected by bats would still be a risk (Griffin et al. 1960, Reiskind and Wund 2009). While we were not able to assess whether wind direction and velocity influenced mosquito flight in the current study, wind is an important variable that should be formally evaluated in future studies involving post prandial movement (Huestis et al. 2019).

As in every study, there were limitations that we made a conscious effort to overcome. Although we sought to accurately identify the nearest locations of each livestock species detected in bloodmeals, we may have missed the locations of some livestock, which would have contributed inaccuracy to our flight distance estimates. However, we are confident in the nearest animal locations we’ve presented. An additional limitation involved host identifications that could not be made with the methods used. Of the 95 blood fed mosquitoes processed, we could only confirm the host identify for 72 (76%). This is common in blood-meal studies as reported by others (Molaei et al. 2008, Kent et al. 2009, Tuten et al. 2012, Reisen et al. 2013) as often the blood is too degraded to be useful for PCR. To improve our chances of host identification, we used known primer sets as others have reported (Kent et al. 2009).

## Conclusions

In conclusion, this study highlights the need for more in-depth studies of mosquito blood feeding within small urban areas to understand how mosquito species interacting with different host species may impact the potential transmission of different pathogens. These small urban areas dominate the landscape of the United States, comprising 76% of the urban areas in the United States (U.S. Census Bureau 2020). By determining what hosts mosquitoes are feeding on in small urban areas, this study highlighted that mosquitoes may be choosing to feed on wildlife and livestock species when they are searching for a bloodmeal. Given the high numbers of mosquitoes which fed on WTD and the proximity of cows and sheep around human habitations, the potential exists for the spread of arboviruses (ie Cache Valley Virus, Rift Valley Fever virus) as several mosquito species collected are known to be susceptible (Andreadis et al. 2014, Golnar et al. 2014, Wilson et al. 2018, Dieme et al. 2022). This study also highlighted that recently blood-fed mosquitoes are able to fly long distances within a short time after feeding. This long-distance post-prandial movement off-sets current ideas that mosquitoes forage on a wide range of hosts within defined areas because of the proximity to good oviposition sites (Reiskind et al. 2017). If mosquitoes forage long distances from larval habitats and freely fly long distances to sheltered sites after blood-feeding, this could mean the associated costs of predation and exertion are worth the increase in potential host choice or some yet to be defined aspect. The importance of post-prandial flight is understudied in vector studies and could be enhanced by marking mosquitoes using stable isotopes in their breeding areas (Hamer et al. 2012, Medeiros et al. 2017). While most WUI studies have focused on exposure of disease vectors in large urban centers (Bisanzio et al. 2020, Kache et al. 2022), the unique environments created for studying the WUI involving mosquitoes and their blood-meal hosts within small ubiquitous urban areas across the United States need further attention.

## Supplementary Material

tjaf105_Supplementary_Data
