# Supplementary material for: Mosquitoes in small urban spaces: identification of blood meals and flight distances of engorged females in the southern Great Plains of the United States
Source: J Med Entomol. 2025 Aug 23;62(5):1200–7. doi: 10.1093/jme/tjaf105 (PMC12507432; doi:10.1093/jme/tjaf105)
Supplement: tjaf105_Supplementary_Data [file tjaf105_supplementary_data.zip › tjaf105_Supplementary_Data/Supp Table 1 Abundance central OK collections.docx]

**Supplemental Table 1:** Total numbers of each mosquito species by trap type collected between June and July 2021 across four exurban trapping locations in central Oklahoma.

| Species | Location | | | | | Trap Type | | |
| --- | --- | --- | --- | --- | --- | --- | --- | --- |
|  | Briarcreek | HighlandP | SangreR | Whittenberg | **Total** | CDC | Bucket | **Total** |
| *Ae. albopictus* | 65 | 52 | 5 | 18 | **140** | 133 | 7 | **140** |
| *Ae. atlanticus* | 0 | 2 | 0 | 0 | **2** | 1 | 1 | **2** |
| *Ae. c. mathesoni* | 0 | 1 | 0 | 0 | **1** | 0 | 1 | **1** |
| *Ae. canadensis* | 0 | 7 | 1 | 0 | **8** | 0 | 8 | **8** |
| *Ae. epactius* | 1 | 0 | 0 | 0 | **1** | 0 | 1 | **1** |
| *Ae. sollicitans* | 0 | 0 | 0 | 1 | **1** | 0 | 1 | **1** |
| *Ae. triseriatus* | 7 | 6 | 0 | 1 | **14** | 9 | 5 | **14** |
| *Ae. trivitattus* | 27 | 144 | 0 | 2 | **173** | 173 | 0 | **173** |
| *Ae. vexans* | 5 | 4 | 0 | 16 | **25** | 15 | 10 | **25** |
| *An. perplexens* | 0 | 0 | 0 | 1 | **1** | 0 | 1 | **1** |
| *An. quadrimaculatus* | 176 | 4 | 9 | 1 | **190** | 0 | 190 | **190** |
| *An. punctipennis* | 26 | 4 | 1 | 4 | **35** | 2 | 33 | **35** |
| *Cx. erraticus* | 110 | 40 | 13 | 20 | **183** | 146 | 37 | **183** |
| *Cx. nigripalpus* | 2 | 1 | 0 | 0 | **3** | 0 | 3 | **3** |
| *Cx. pipiens* | 8 | 10 | 1 | 4 | **23** | 5 | 18 | **23** |
| *Cx. restuans* | 0 | 1 | 0 | 0 | **1** | 1 | 0 | **1** |
| *Cx. salinarius* | 2 | 1 | 0 | 3 | **6** | 6 | 0 | **6** |
| *Cx. tarsalis* | 3 | 1 | 1 | 2 | **7** | 5 | 2 | **7** |
| *Ps. ciliata* | 0 | 1 | 0 | 0 | **1** | 1 | 0 | **1** |
| *Ps. columbiae* | 2 | 7 | 6 | 21 | **36** | 31 | 5 | **36** |
| *Ps. cyanescens* | 3 | 20 | 3 | 0 | **26** | 21 | 5 | **26** |
| *Ps. ferox* | 3 | 25 | 1 | 1 | **30** | 24 | 6 | **30** |
| *Ps. longipalpus* | 3 | 13 | 0 | 0 | **16** | 16 | 0 | **16** |
| *Unknown* | 0 | 2 | 0 | 0 | **2** | 2 | 0 | **2** |
| Total | 443 | 346 | 41 | 95 | **925** | 591 | 334 | **925** |

HighlandP: Highland Park Elementary School, SangreR: Sangre Ridge, Whittenberg: Whittenberg Park
