# Supplementary material for: Mosquitoes in small urban spaces: identification of blood meals and flight distances of engorged females in the southern Great Plains of the United States
Source: J Med Entomol. 2025 Aug 23;62(5):1200–7. doi: 10.1093/jme/tjaf105 (PMC12507432; doi:10.1093/jme/tjaf105)
Supplement: tjaf105_Supplementary_Data [file tjaf105_supplementary_data.zip › tjaf105_Supplementary_Data/Supplemental_Figures_1-4_revised.docx]

**Supplemental Figures 1-4**


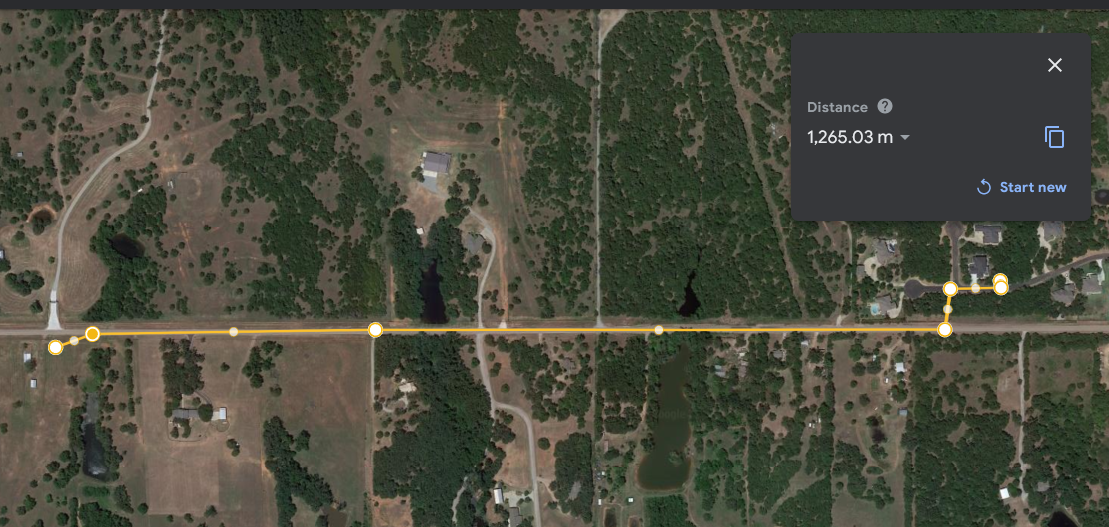


**A**

**Alpaca site**

**Trap site**


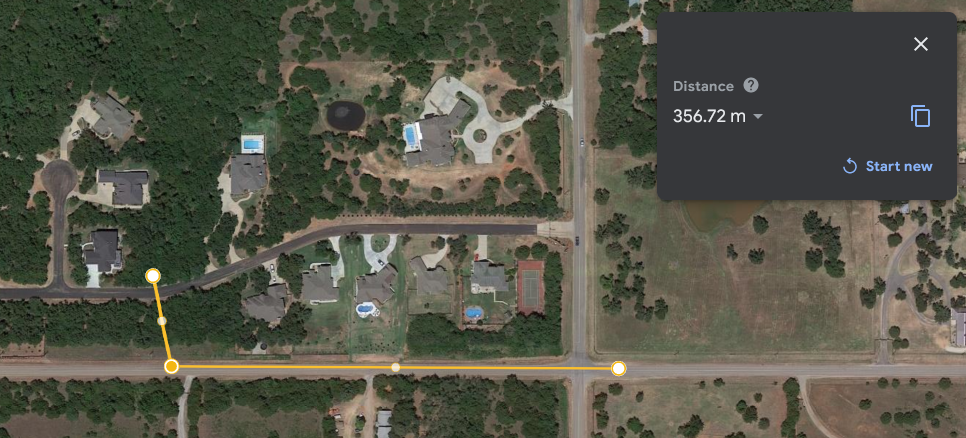


**B**

**Cattle and sheep sites**

**Trap site**

Supplemental Figure 1. Minimum distance between the closest site location of particular animals and the resting trap in which the blood-fed mosquito was collected at the collection location. A) Briar Creek – west alpaca site; B) Briar Creek – eastern cattle and sheep sites. (Google Earth)


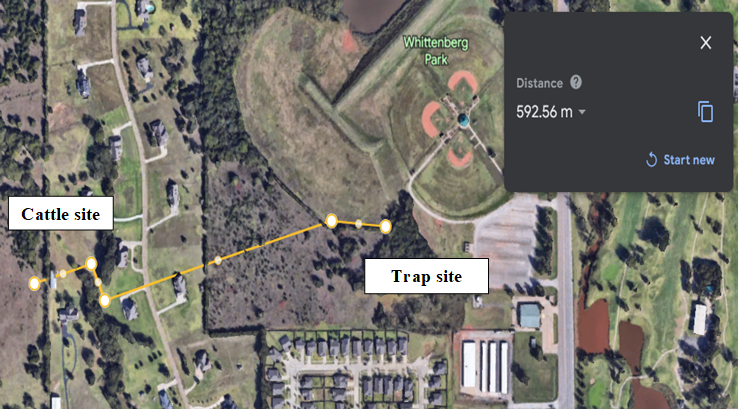
Supplemental Figure 2. Minimum distance between the closest site location of cattle and the resting trap in which the blood-fed mosquito was collected at Whittenberg Park (Google Earth).

Supplemental Figure 3. Minimum distance between the closest site location of horse and the resting trap in which the blood-fed mosquito was collected at Sangre Ridge location (Google Earth).**
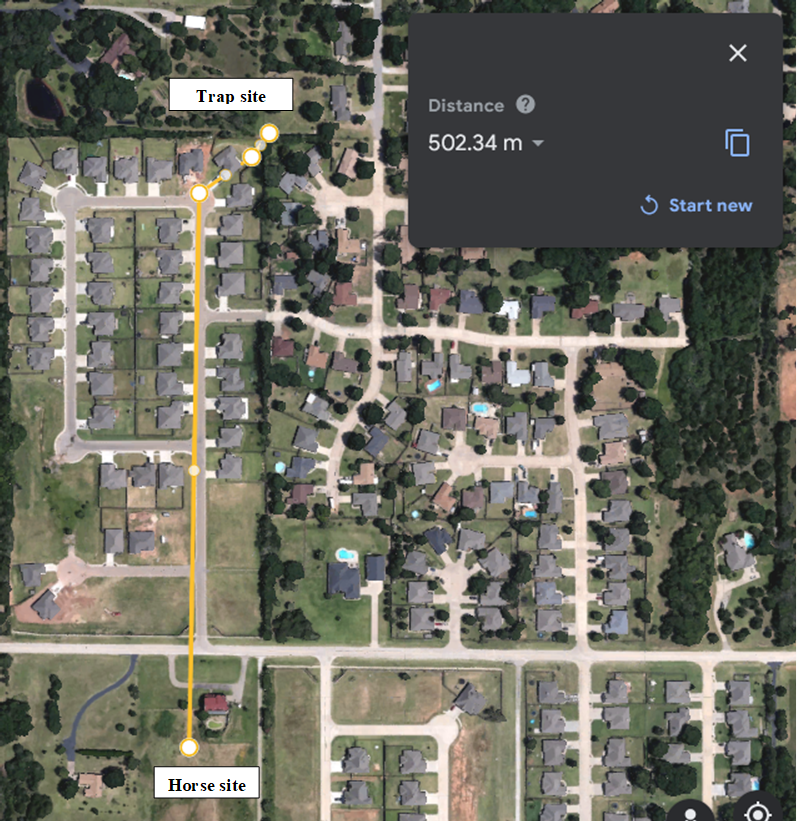
**


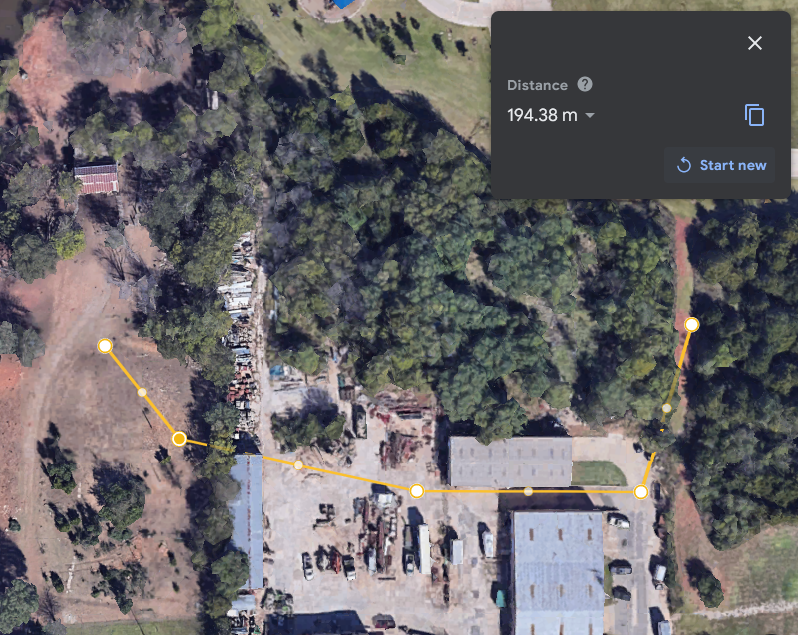


Supplemental Figure 4. Minimum distance between the closest site location of horse and the resting trap in which the blood-fed mosquito was collected at Highland Park location (Google Earth).
